# 576 Pixel-Grafting and Negative-Pressure Moist Wound Chambers Improve Re-epithelization After Burn Injuries

**DOI:** 10.1093/jbcr/irad045.171

**Published:** 2023-05-15

**Authors:** Yadira Villalvazo, Pooja Humar, Fuat Baris Bengur, Shawn J Loder, Wayne V Nerone, Alexandra Vagonis, Bahaa Shaaban, Lauren E Kokai, Kacey G Marra, Elof Eriksson, J Peter Rubin

**Affiliations:** University of Pittsburgh Department of Plastic Surgery, Pittsburgh, Pennsylvania; University of Pittsburgh Department of Plastic Surgery, Pittsburgh, Pennsylvania; University of Pittsburgh Department of Plastic Surgery, Pittsburgh, Pennsylvania; University of Pittsburgh Department of Plastic Surgery, Pittsburgh, Pennsylvania; University of Pittsburgh Department of Plastic Surgery, Pittsburgh, Pennsylvania; Unviersity of Pittsburgh Department of Plastic Surgery, Pittsburgh, Pennsylvania; University of Pittsburgh Department of Plastic Surgery, Pittsburgh, Pennsylvania; Unviersity of Pittsburgh Department of Plastic Surgery, Pittsburgh, Pennsylvania; University of Pittsburgh Department of Plastic Surgery, Pittsburgh, Pennsylvania; Harvard Medical School, Boston, Massachusetts; University of Pittsburgh Department of Plastic Surgery, Pittsburgh, Pennsylvania; University of Pittsburgh Department of Plastic Surgery, Pittsburgh, Pennsylvania; University of Pittsburgh Department of Plastic Surgery, Pittsburgh, Pennsylvania; University of Pittsburgh Department of Plastic Surgery, Pittsburgh, Pennsylvania; University of Pittsburgh Department of Plastic Surgery, Pittsburgh, Pennsylvania; University of Pittsburgh Department of Plastic Surgery, Pittsburgh, Pennsylvania; Unviersity of Pittsburgh Department of Plastic Surgery, Pittsburgh, Pennsylvania; University of Pittsburgh Department of Plastic Surgery, Pittsburgh, Pennsylvania; Unviersity of Pittsburgh Department of Plastic Surgery, Pittsburgh, Pennsylvania; University of Pittsburgh Department of Plastic Surgery, Pittsburgh, Pennsylvania; Harvard Medical School, Boston, Massachusetts; University of Pittsburgh Department of Plastic Surgery, Pittsburgh, Pennsylvania; University of Pittsburgh Department of Plastic Surgery, Pittsburgh, Pennsylvania; University of Pittsburgh Department of Plastic Surgery, Pittsburgh, Pennsylvania; University of Pittsburgh Department of Plastic Surgery, Pittsburgh, Pennsylvania; University of Pittsburgh Department of Plastic Surgery, Pittsburgh, Pennsylvania; University of Pittsburgh Department of Plastic Surgery, Pittsburgh, Pennsylvania; Unviersity of Pittsburgh Department of Plastic Surgery, Pittsburgh, Pennsylvania; University of Pittsburgh Department of Plastic Surgery, Pittsburgh, Pennsylvania; Unviersity of Pittsburgh Department of Plastic Surgery, Pittsburgh, Pennsylvania; University of Pittsburgh Department of Plastic Surgery, Pittsburgh, Pennsylvania; Harvard Medical School, Boston, Massachusetts; University of Pittsburgh Department of Plastic Surgery, Pittsburgh, Pennsylvania; University of Pittsburgh Department of Plastic Surgery, Pittsburgh, Pennsylvania; University of Pittsburgh Department of Plastic Surgery, Pittsburgh, Pennsylvania; University of Pittsburgh Department of Plastic Surgery, Pittsburgh, Pennsylvania; University of Pittsburgh Department of Plastic Surgery, Pittsburgh, Pennsylvania; University of Pittsburgh Department of Plastic Surgery, Pittsburgh, Pennsylvania; Unviersity of Pittsburgh Department of Plastic Surgery, Pittsburgh, Pennsylvania; University of Pittsburgh Department of Plastic Surgery, Pittsburgh, Pennsylvania; Unviersity of Pittsburgh Department of Plastic Surgery, Pittsburgh, Pennsylvania; University of Pittsburgh Department of Plastic Surgery, Pittsburgh, Pennsylvania; Harvard Medical School, Boston, Massachusetts; University of Pittsburgh Department of Plastic Surgery, Pittsburgh, Pennsylvania; University of Pittsburgh Department of Plastic Surgery, Pittsburgh, Pennsylvania; University of Pittsburgh Department of Plastic Surgery, Pittsburgh, Pennsylvania; University of Pittsburgh Department of Plastic Surgery, Pittsburgh, Pennsylvania; University of Pittsburgh Department of Plastic Surgery, Pittsburgh, Pennsylvania; University of Pittsburgh Department of Plastic Surgery, Pittsburgh, Pennsylvania; Unviersity of Pittsburgh Department of Plastic Surgery, Pittsburgh, Pennsylvania; University of Pittsburgh Department of Plastic Surgery, Pittsburgh, Pennsylvania; Unviersity of Pittsburgh Department of Plastic Surgery, Pittsburgh, Pennsylvania; University of Pittsburgh Department of Plastic Surgery, Pittsburgh, Pennsylvania; Harvard Medical School, Boston, Massachusetts; University of Pittsburgh Department of Plastic Surgery, Pittsburgh, Pennsylvania; University of Pittsburgh Department of Plastic Surgery, Pittsburgh, Pennsylvania; University of Pittsburgh Department of Plastic Surgery, Pittsburgh, Pennsylvania; University of Pittsburgh Department of Plastic Surgery, Pittsburgh, Pennsylvania; University of Pittsburgh Department of Plastic Surgery, Pittsburgh, Pennsylvania; University of Pittsburgh Department of Plastic Surgery, Pittsburgh, Pennsylvania; Unviersity of Pittsburgh Department of Plastic Surgery, Pittsburgh, Pennsylvania; University of Pittsburgh Department of Plastic Surgery, Pittsburgh, Pennsylvania; Unviersity of Pittsburgh Department of Plastic Surgery, Pittsburgh, Pennsylvania; University of Pittsburgh Department of Plastic Surgery, Pittsburgh, Pennsylvania; Harvard Medical School, Boston, Massachusetts; University of Pittsburgh Department of Plastic Surgery, Pittsburgh, Pennsylvania; University of Pittsburgh Department of Plastic Surgery, Pittsburgh, Pennsylvania; University of Pittsburgh Department of Plastic Surgery, Pittsburgh, Pennsylvania; University of Pittsburgh Department of Plastic Surgery, Pittsburgh, Pennsylvania; University of Pittsburgh Department of Plastic Surgery, Pittsburgh, Pennsylvania; University of Pittsburgh Department of Plastic Surgery, Pittsburgh, Pennsylvania; Unviersity of Pittsburgh Department of Plastic Surgery, Pittsburgh, Pennsylvania; University of Pittsburgh Department of Plastic Surgery, Pittsburgh, Pennsylvania; Unviersity of Pittsburgh Department of Plastic Surgery, Pittsburgh, Pennsylvania; University of Pittsburgh Department of Plastic Surgery, Pittsburgh, Pennsylvania; Harvard Medical School, Boston, Massachusetts; University of Pittsburgh Department of Plastic Surgery, Pittsburgh, Pennsylvania; University of Pittsburgh Department of Plastic Surgery, Pittsburgh, Pennsylvania; University of Pittsburgh Department of Plastic Surgery, Pittsburgh, Pennsylvania; University of Pittsburgh Department of Plastic Surgery, Pittsburgh, Pennsylvania; University of Pittsburgh Department of Plastic Surgery, Pittsburgh, Pennsylvania; University of Pittsburgh Department of Plastic Surgery, Pittsburgh, Pennsylvania; Unviersity of Pittsburgh Department of Plastic Surgery, Pittsburgh, Pennsylvania; University of Pittsburgh Department of Plastic Surgery, Pittsburgh, Pennsylvania; Unviersity of Pittsburgh Department of Plastic Surgery, Pittsburgh, Pennsylvania; University of Pittsburgh Department of Plastic Surgery, Pittsburgh, Pennsylvania; Harvard Medical School, Boston, Massachusetts; University of Pittsburgh Department of Plastic Surgery, Pittsburgh, Pennsylvania; University of Pittsburgh Department of Plastic Surgery, Pittsburgh, Pennsylvania; University of Pittsburgh Department of Plastic Surgery, Pittsburgh, Pennsylvania; University of Pittsburgh Department of Plastic Surgery, Pittsburgh, Pennsylvania; University of Pittsburgh Department of Plastic Surgery, Pittsburgh, Pennsylvania; University of Pittsburgh Department of Plastic Surgery, Pittsburgh, Pennsylvania; Unviersity of Pittsburgh Department of Plastic Surgery, Pittsburgh, Pennsylvania; University of Pittsburgh Department of Plastic Surgery, Pittsburgh, Pennsylvania; Unviersity of Pittsburgh Department of Plastic Surgery, Pittsburgh, Pennsylvania; University of Pittsburgh Department of Plastic Surgery, Pittsburgh, Pennsylvania; Harvard Medical School, Boston, Massachusetts; University of Pittsburgh Department of Plastic Surgery, Pittsburgh, Pennsylvania; University of Pittsburgh Department of Plastic Surgery, Pittsburgh, Pennsylvania; University of Pittsburgh Department of Plastic Surgery, Pittsburgh, Pennsylvania; University of Pittsburgh Department of Plastic Surgery, Pittsburgh, Pennsylvania; University of Pittsburgh Department of Plastic Surgery, Pittsburgh, Pennsylvania; University of Pittsburgh Department of Plastic Surgery, Pittsburgh, Pennsylvania; Unviersity of Pittsburgh Department of Plastic Surgery, Pittsburgh, Pennsylvania; University of Pittsburgh Department of Plastic Surgery, Pittsburgh, Pennsylvania; Unviersity of Pittsburgh Department of Plastic Surgery, Pittsburgh, Pennsylvania; University of Pittsburgh Department of Plastic Surgery, Pittsburgh, Pennsylvania; Harvard Medical School, Boston, Massachusetts; University of Pittsburgh Department of Plastic Surgery, Pittsburgh, Pennsylvania; University of Pittsburgh Department of Plastic Surgery, Pittsburgh, Pennsylvania; University of Pittsburgh Department of Plastic Surgery, Pittsburgh, Pennsylvania; University of Pittsburgh Department of Plastic Surgery, Pittsburgh, Pennsylvania; University of Pittsburgh Department of Plastic Surgery, Pittsburgh, Pennsylvania; University of Pittsburgh Department of Plastic Surgery, Pittsburgh, Pennsylvania; Unviersity of Pittsburgh Department of Plastic Surgery, Pittsburgh, Pennsylvania; University of Pittsburgh Department of Plastic Surgery, Pittsburgh, Pennsylvania; Unviersity of Pittsburgh Department of Plastic Surgery, Pittsburgh, Pennsylvania; University of Pittsburgh Department of Plastic Surgery, Pittsburgh, Pennsylvania; Harvard Medical School, Boston, Massachusetts; University of Pittsburgh Department of Plastic Surgery, Pittsburgh, Pennsylvania

## Abstract

**Introduction:**

Complex burns are a challenge, often requiring prolonged reconstruction. Management requires consideration not only to the skin but to underlying structures and often requires staged-reconstruction and revision for increasingly unsatisfactory results. This standard is expensive, limited by donor-site availability, and often impacts quality of life. Here we demonstrate the efficacy of single-staged minced skin grafting in an incubator-like microenvironment with a negative-pressure moist wound chamber device to achieve rapid epithelialization after burns.

**Methods:**

Full-thickness burns were induced to female Yorkshire swine. Escharectomies were performed to the level of fascia after seventy-two hours. One group received standard of care skin grafting with a bolster dressing. In another group, split-thickness skin was cut into pixel size (0.3x0.3 mm) grafts, followed by application of either bolster or negative-pressure moist wound chamber dressing. Wounds were followed for 4-weeks with serial photography, ultrasound, and biopsies for histology.

**Results:**

As early as one week, epithelialization started in the group with pixel grafts with negative-pressure moist wound chamber dressings with visible epithelioid islands on the wound bed granulation tissue. This progressed with a similar trend throughout the 4-week period eventually leading to near-complete epithelization and keratinization. There was reconstruction of trilaminar cutaneous architecture demonstrated by the presence of distinct, viable epidermal, dermal and hypodermal elements as well as viability of adipose on histology. Distinct differences in contour were noted between the bolster and negative-pressure moist wound chamber groups.

**Conclusions:**

Minced grafting minimized donor burden and alleviated the need for graft orientation. In both the bolstered and negative-pressure moist wound chambers, pixel-grafts viably survived to form a viable basal layer. Use of positive-pressure (bolster) vs. negative-pressure moist wound chamber dressings demonstrated distinct differences in the convexity/concavity and topography of the singe-stage skin graft with critical implications for aesthetic reconstruction.

**Applicability of Research to Practice:**

This study introduces the efficacy of a single-staged trilaminar reconstruction with the use of a negative-pressure moist wound chamber for complex burns. These findings support the potential for a new paradigm in the treatment of complex burns which allows for single-stage reconstruction with minimal donor site morbidity.

